# Chemically modified, non-anticoagulant heparin derivatives are potent galectin-3 binding inhibitors and inhibit circulating galectin-3-promoted metastasis

**DOI:** 10.18632/oncotarget.4409

**Published:** 2015-06-23

**Authors:** Carrie A. Duckworth, Scott E. Guimond, Paulina Sindrewicz, Ashley J. Hughes, Neil S. French, Lu-Yun Lian, Edwin A. Yates, D. Mark Pritchard, Jonathan M. Rhodes, Jeremy E. Turnbull, Lu-Gang Yu

**Affiliations:** ^1^ Department of Gastroenterology, Institute of Translational Medicine, University of Liverpool, Liverpool, United Kingdom; ^2^ Department of Biochemistry, Institute of Integrative Biology, University of Liverpool, Liverpool, United Kingdom; ^3^ Department of Molecular and Clinical Pharmacology, Institute of Translational Medicine, University of Liverpool, Liverpool, United Kingdom; ^4^ Diamond Light Source Ltd, Harwell Innovation Campus, Didcot, UK

**Keywords:** galectin-3, metastasis, heparin

## Abstract

Concentrations of circulating galectin-3, a metastasis promoter, are greatly increased in cancer patients. Here we show that 2- or 6-de-*O*-sulfated, *N*-acetylated heparin derivatives are galectin-3 binding inhibitors. These chemically modified heparin derivatives inhibited galectin-3-ligand binding and abolished galectin-3-mediated cancer cell-endothelial adhesion and angiogenesis. Unlike standard heparin, these modified heparin derivatives and their ultra-low molecular weight sub-fractions had neither anticoagulant activity nor effects on E-, L- or P-selectin binding to their ligands nor detectable cytotoxicity. Intravenous injection of such heparin derivatives (with cancer cells pre-treated with galectin-3 followed by 3 subcutaneous injections of the derivatives) abolished the circulating galectin-3-mediated increase in lung metastasis of human melanoma and colon cancer cells in nude mice. Structural analysis using nuclear magnetic resonance and synchrotron radiation circular dichroism spectroscopies showed that the modified heparin derivatives bind to the galectin-3 carbohydrate-recognition domain. Thus, these chemically modified, non-anticoagulant, low-sulfated heparin derivatives are potent galectin-3 binding inhibitors with substantial potential as anti-metastasis/cancer drugs.

## INTRODUCTION

Metastasis is the main cause of cancer mortality and is associated with greater than 90% of all cancer-related deaths. Despite recent advances in the understanding of tumorigenesis, the underlying mechanisms of metastatic dissemination remain less well characterised [1]. Metastatic spread from primary to secondary tumor sites occurs via the blood or lymphatic circulations. Adhesion of disseminating cancer cells to the vascular endothelium and homotypic aggregation of tumor cells that results in circulating tumor micro-emboli, are both essential steps in the metastatic cascade.

Galectin-3 is a galactoside-binding protein that is expressed by various types of human cells. The concentration of galectin-3 is elevated by up to 30-fold in the circulation of cancer patients including those with colon, breast, pancreatic, melanoma, lung, head and neck, and ovarian cancer and non-Hodgkin's lymphoma [2–7]. Patients with metastasis have higher levels of circulating galectin-3 than those with only localized tumors [2]. Recent studies have shown that increased concentrations of circulating galectin-3 promote dissemination of tumour cell metastatic spread to remote sites [8–17]. These effects are partly attributable to its interaction with the oncofetal Galβ1, 3GalNAcα- (TF) antigen expressed on the cancer cell membrane-associated mucin proteins MUC1 [18] and MUC4 [12]. The galectin-3-MUC1/4 interaction induces MUC1/4 cell surface polarization and results in the exposure of smaller cell surface adhesion molecules leading to increased adhesion of disseminating tumor cells to the vascular endothelium, and also increased aggregation of cancer cells resulting in the potential formation of circulating tumor emboli [9, 10]. Circulating galectin-3 also promotes endothelial secretion of metastasis-promoting cytokines [9] and cancer cell migration [13], proliferation [14] and angiogenesis [15, 16]. Several biotech companies have therefore initiated research programmes to develop galectin-3 inhibitors for preventing metastasis. Several have shown promise in animal models and are currently undergoing clinical trials (e.g. GMCT-01 and GRMD-02; GCS-100) [17].

Heparin is a heterogeneous N- and O-sulfated glycosaminoglycan anti-coagulant that is often used for the treatment or prevention of thromboembolism in cancer patients. Previous studies have documented an inhibitory effect of unfractionated heparin (UFH) in animal models of cancer metastasis [19]. Human clinical trials of unfractionated heparin (UFH) in cancer have, however, been mixed, with some showing improved patient survival and others showing no effect [20]. Part of the unpredictability is believed to derive from the “non-specific” effects of UFH on a wide variety of cellular processes as a result of its interactions with many plasma and cellular proteins due in part to its high charge [21], and its ability to mimic heparan sulphate and interact with complex signalling networks [22]. Up to 22% of all plasma proteins have been shown to be extractable by UFH affinity purification [23]. In addition, limitations on dosage imposed by the anticoagulant activity of heparin restrict its therapeutic efficacy as an anti-metastatic agent. Recent clinical trials have focussed on use of low molecular weight heparins (LMWH) in cancer and have suggested possible survival benefit [21]. Chemically modified heparins of lower molecular weight and altered charge may have enhanced therapeutic ratios and bioavailability, whilst exhibiting reduced off-target effects (such as reduced anticoagulation properties) [24].

In a search for potential anti-metastasis agents that target the actions of circulating galectin-3, several chemically-modified (selectively de-sulfated) heparin derivatives were screened and 2- or 6-de-O-sulfated heparin derivatives were identified as potent galectin-3-binding inhibitors. These chemically-modified heparin derivatives, which show no detectable anti-coagulant activity and cytotoxicity, bind to galectin-3 carbohydrate recognition domain (CRD) and inhibit galectin-3-mediated cancer cell adhesion and angiogenesis *in vitro* and metastasis *in vivo* in a mouse model.

## RESULTS

### Selectively de-sulfated, N-acetylated heparin derivatives inhibit galectin-3 binding to TF-expressing asialo-fetuin

**Figure 1:**
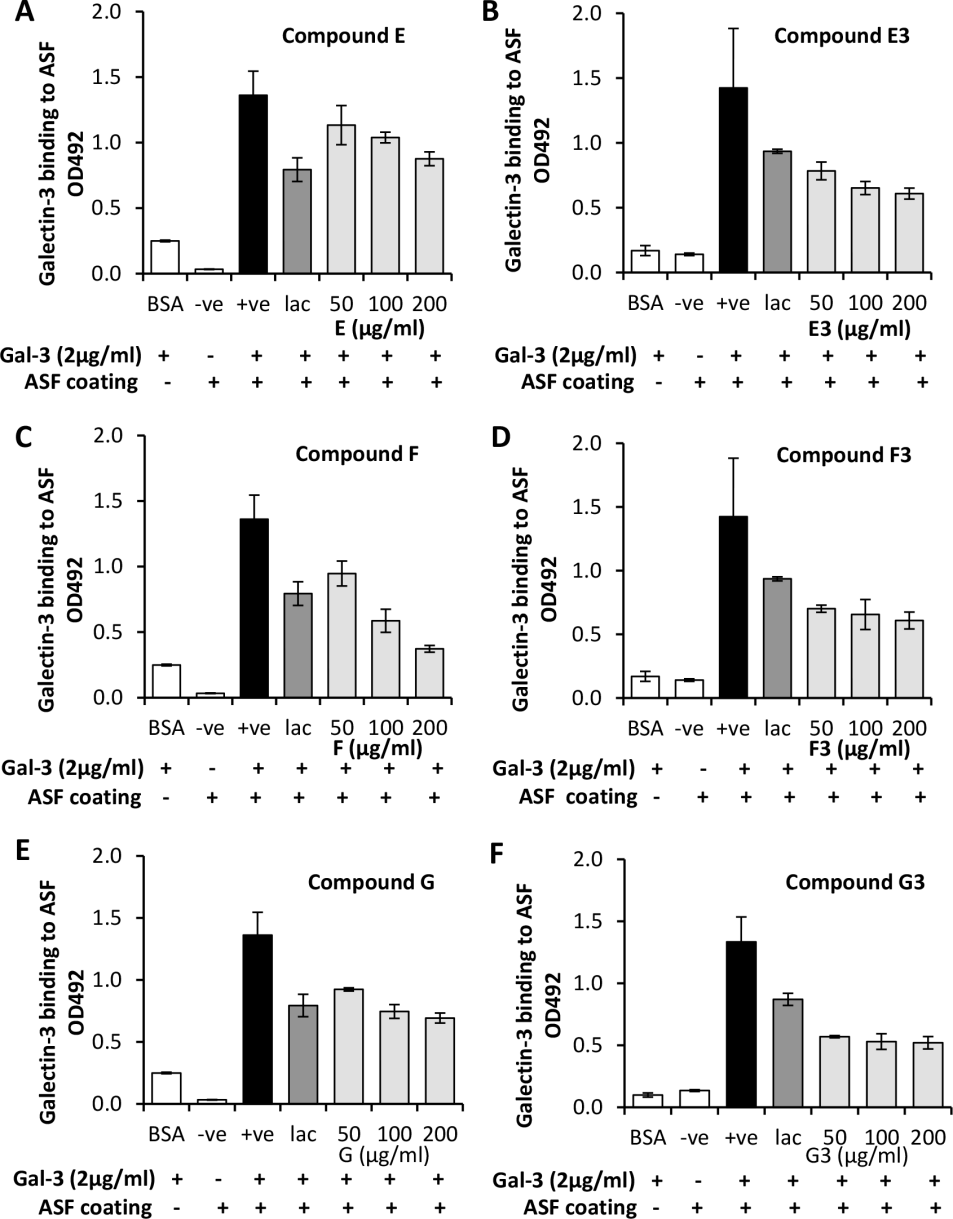
Modified heparin derivatives inhibit galectin-3 binding to TF-expressing glycan The impact of the derivatives E **(panel A)**, F **(panel C)** and G **(panel E)** and ultra-low molecular weight fractions E3 **(panel B)**, F3 **(panel D)** and G3 **(panel F)** on galectin-3 binding to TF-expressing asialofetuin were assessed by ELISA. Galectin-3 binding to BSA was used as a negative binding control in addition to incubation of wells with no galectin-3. Lactose (lac) was added as a positive inhibition control.

Twenty-four heparin derivatives produced by selective chemical modification and size fractionation (Supplementary Table S3) were screened for their effects on galectin-3 binding to the TF-expressing asialo-fetuin (Supplementary Fig. S2). 2 or 6-de-sulfated, *N*-acetylated heparin derivatives (E and F) and 2- and 6-de-*O*-sulfated, *N*-sulfated heparin (G) produced significant inhibition of galectin-3 binding to asialo-fetuin (Fig. 1A–1F). Note that this binding inhibition was not observed by fully sulfated unfractionated and unmodified heparin (Supplementary Fig. S2). The ultra-low molecular weight sub-fractions of these derivatives (<3000 Da; E3, F3 and G3) seem to be more effective inhibitors of galectin-3 binding than the unfractionated compounds on a weight basis. For example, compound E showed a 20.0 ± 2.6% inhibition of galectin-3 binding at a dose of 50 μg/ml, while its lower molecular weight sub-fraction (E3) resulted in 44.9 ± 3.9% inhibition at this concentration. Although there may be little apparent difference between the inhibitory potency of fractionated and unfractionated compounds on a simple molar basis, comparisons on a weight basis may in fact more accurately reflect available binding sites on these polymeric molecules. When tested for their influence on galectin-1, -4 and -8 binding to asialo-feutin under similar conditions, none of the galectin-3-binding inhibitory heparin derivatives showed significant inhibition of binding by these galectins (Supplementary Fig. S3), suggesting significant specificity for galectin-3 compared to other galectin family members.

### Low sulfated heparin derivatives inhibit circulating galectin-3-mediated cancer cell adhesion to endothelial monolayers

**Figure 2:**
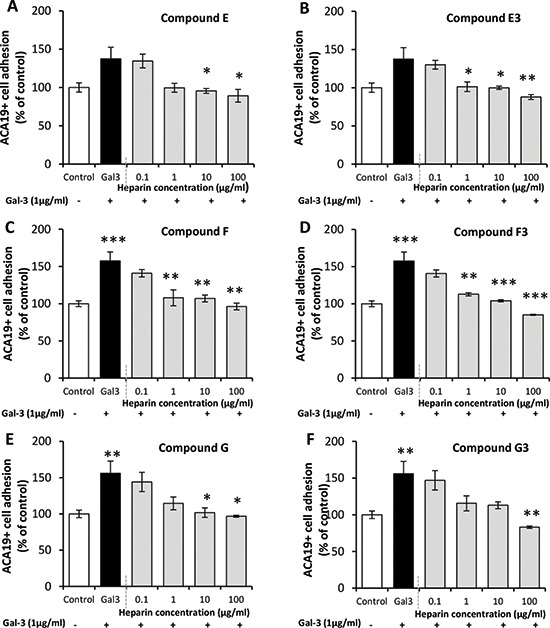
Modified heparin derivatives inhibit galectin-3-mediated cancer cell adhesion to endothelial cells The presence of compounds E **(panel A)**, F **(panel C)** and G **(panel E)** and ultra-low molecular weight fractions E3 **(panel B)**, F3 **(panel D)** and G3 **(panel F)** each prevented galectin-3-mediated adhesion of ACA19+ cells to a HUVEC monolayer compared with BSA treated control. ***p < 0.001, **p < 0.01, *p < 0.05 by one-way ANOVA, three independent experiments each in triplicate.

Increased cancer cell adhesion to vascular endothelium is one of the important metastasis-promoting effects of circulating galectin-3. As heparin derivatives E, E3, F, F3, G and G3 each showed inhibition of galectin-3 ligand-binding, their influence on galectin-3-mediated cancer cell adhesion to primary endothelial cells was assessed. Pre-treatment of MUC1-expressing human melanoma ACA19+ cells with galectin-3 before application of the cells to HUVEC monolayers resulted in a ∼50% increase of cell adhesion in comparison to the BSA-treated control cells. Pre-incubation of galectin-3 with E, E3, F, F3, G or G3 followed by incubation with ACA19+ and subsequent application of the cells to HUVEC monolayers resulted in inhibition of galectin-3-mediated cell adhesion in a dose-dependent manner (Fig. 2).

### The low sulfated heparin derivatives have no detectable anticoagulant activity, or cytotoxicity and no effect on selectin binding

The effects of these heparin derivatives on the three (intrinsic, extrinsic and common) pathways of the clotting cascade were measured. None of the heparin derivatives E, F and G or their subfractions showed any anticoagulation activity, either in general assays for APTT (activated partial thromboplastin time) or PT (prothrombin time) activity, or specifically on the activities of Factor IIa or Factor Xa (Table 1). Of the other derivatives, D and D1 showed anticoagulation activity, in the APTT assay only, with 3 fold less activity than unfractionated heparin.

**Table 1.** The modified heparin derivatives show no detectable anticoagulant activity compared to standard heparin

Compounds	Structural modifications	Factor XaIC50 (μg/ml)	Factor IIaIC50 (μg/ml)	APTT IC50 (μg/ml)	PT IC50 (μg/ml)
**Heparin**		0.93	1.03	1.25	41.40
**E**	2-de-O-sulfated, N-acetylated	NI^a^	NI	NI	NI
**E3**	2-de-O-sulfated, N-acetylated <3000 KDa	NI	NI	NI	NI
**F**	6-de-O-sulfated, N-acetylated	NI	NI	NI	NI
**F3**	6-de-O-sulfated, N-acetylated <3000 kDa	NI	NI	NI	NI
**G**	2, 6-de-O-sulfated	NI	NI	NI	NI
**G3**	2, 6-de-O-sulfated <3000 kDa	NI	NI	NI	NI

a NI, no inhibition of coagulation up to 100 μg/ml compounds

Inhibition of selectin binding and hence of leucocyte recruitment is another potential problem with standard heparins used for metastasis prevention, so the effects of the modified heparin derivatives on binding of E-, L-, and P- selectins to their ligand sialyl Lewis^x^ were also tested. None of the heparin derivatives showed detectable interference with selectin binding to sialyl Lewis^x^ (Table 2). Furthermore, none of the derivatives showed any detectable cytotoxicity to cells when they were treated at 100 μg/ml for 24 or 48 hr (Supplementary Table S4).

**Table 2.** The modified heparin derivatives show no detectable effect on L-selectin, P-selectin and E-selectin binding to sialyl-Lewis^x^ compared to standard heparin

Compounds	Structural modifications	L-selectinIC50 (μg/ml)	P-selectinIC50 (μg/ml)	E-selectinIC50 (μg/ml)
**Heparin**		1.63	4.0	NI^a^
**E**	2-de-O-sulfated, N-acetylated	NI^a^	NI	NI
**E3**	2-de-O-sulfated, N-acetylated < 3000KDa	NI	NI	NI
**F**	6-de-O-sulfated, N-acetylated	NI	NI	NI
**F3**	6-de-O-sulfated, N-acetylated < 3000kDa	NI	NI	NI
**G**	2, 6-de-O-sulfated	NI	NI	NI
**G3**	2, 6-de-O-sulfated < 3000kDa	NI	NI	NI

a NI, no inhibition of selectin binding up to 100 μg/ml compounds

### The low sulfated heparin derivatives inhibit circulating galectin-3-mediated increase of lung metastasis in mice

**Figure 3:**
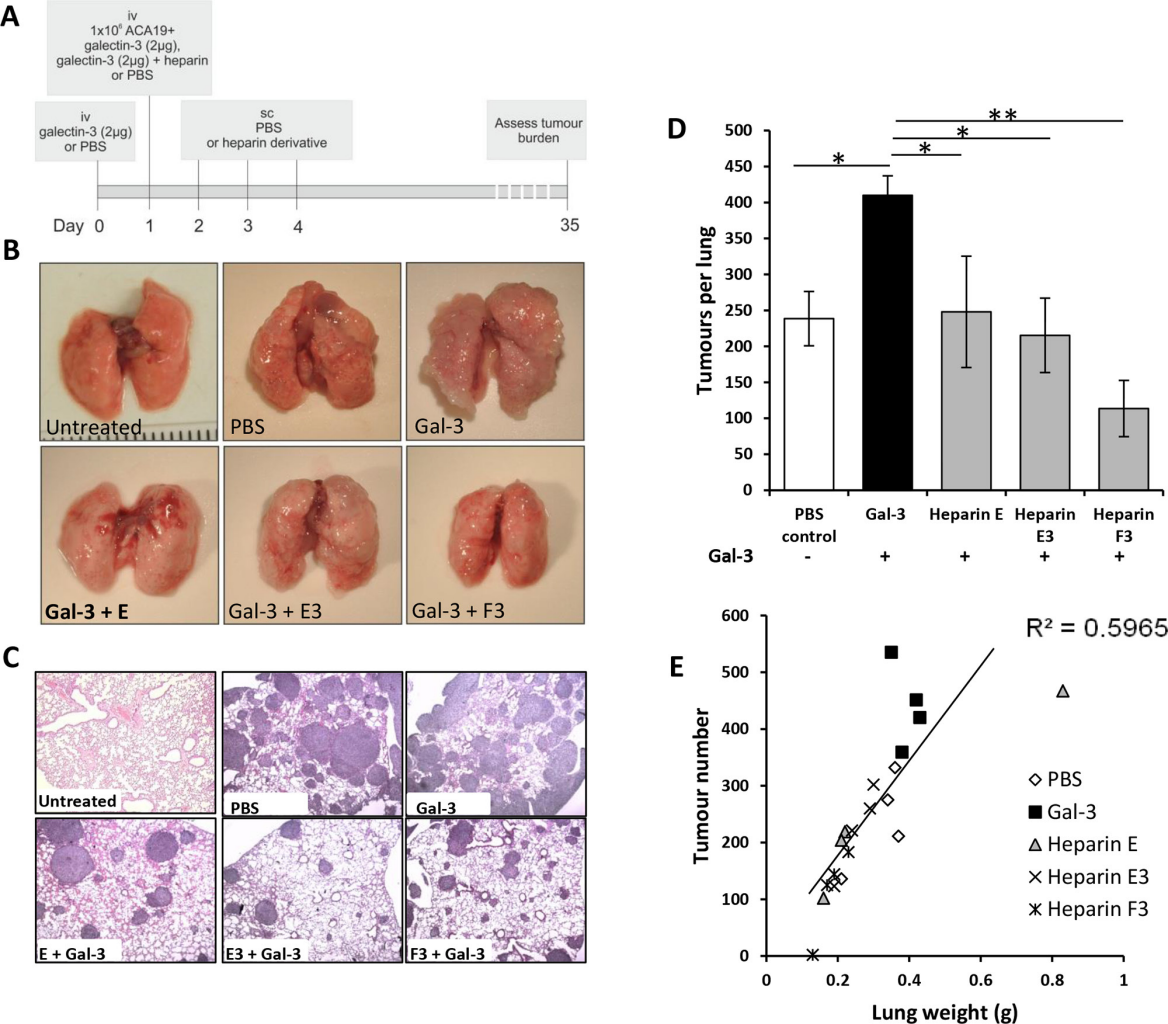
Modified heparin derivatives prevent galectin-3 mediated metastasis of human melanoma ACA19+ cells in nude mice Schematic representation of the experimental protocol **A.** Gross images of lungs **B.** or H and E stained photomicrographs **C.** from Balb/c nude mice administered with ACA19+ melanoma cells iv and also co-injected with galectin-3 with or without heparin derivatives E, E3, F, F3, G or G3 (20 mg/kg) iv. Mean tumors per lung **D.** and lung weight vs tumor number **E.** are shown for all experimental groups. *p < 0.05 by one-way ANOVA, *n* = 4 mice per experimental group.

As the heparin derivatives E, E3 and F3 showed significant inhibition of galectin-3-mediated cancer cell-endothelial adhesion, these derivatives were tested for their impact on circulating galectin-3-mediated metastasis in an experimental mouse metastasis model. Earlier studies have shown that the impact of circulating galectin-3 on MUC1-polarization and cell adhesion occurs within 1 hour while its influence on endothelial secretion of metastasis-promoting cytokines takes over 20 hr [9–11]. The experiments were therefore designed to capture both the short-term and longer-term effects of circulating galectin-3 on metastasis. The mice were injected via the tail vein with 2 μg galectin-3, equivalent to 1.25 μg/ml blood (assuming 1.6 ml total blood volume), a pathological circulating galectin-3 concentration found in patients with metastatic cancer, or PBS to control mice, 24 hr prior to the administration of tumor cells to prime the mice. ACA19+ cells were then administered by tail vein injection in combination with galectin-3 with or without each of the heparin derivatives. Three more administrations of the heparin derivatives were subsequently given daily by subcutaneous injection (Fig. 3A). The animals were sacrificed 5 weeks after tumor cell injection and organs examined for metastases.

Metastasis was found to occur exclusively in the lungs (no metastatic foci were observed in brain, liver, kidney, spleen, stomach, colon, small intestine or heart). In comparison to the control group (238 ± 42 tumor nodules per lung), the animals in the galectin-3-treated group showed significantly more metastatic nodules (437 ± 36 tumor nodules, *p* < 0.05) assessed by surface inspection after blind labelling using a dissecting microscope (Fig. 3B–3E). Significant reductions in tumor numbers per lung, and lung weights were observed in the group of animals that were treated with heparin derivatives E (95 ± 38% reduction in galectin-3 induced metastasis, *p* = 0.001), E3 (106 ± 19% reduction in galectin-3 induced metastasis, *p* < 0.05) and F3 (161 ± 19% reduction in galectin-3 induced metastasis, *p* < 0.01) in comparison to the galectin-3 treated group (0 ± 18% reduction) (Fig. 3D and 3E). A good positive correlation (R^2^ = 0.6) between lung weight and tumor number was observed across all treatment groups (Fig. 3E). There was no significant difference in tumor nodule diameter measured from H and E stained sections between any of the groups although data showed a tendency towards reduced tumor diameter in E3 and F3 treated groups (data not shown). There was also no significant difference of change of animal body weights among the animal groups during the experimental period (Supplementary Fig. S4A), suggesting these heparin derivatives, like the standard heparin, have no apparent toxicity. Notably F3 not only abolished the circulating galectin-3-induced increase in metastasis as judged by lung weight, but also caused a significant additional reduction in metastasis compared to the control (control, 0.32 ± 0.03 g; F3, 0.18 ± 0.02 g; *p* < 0.05).

**Figure 4:**
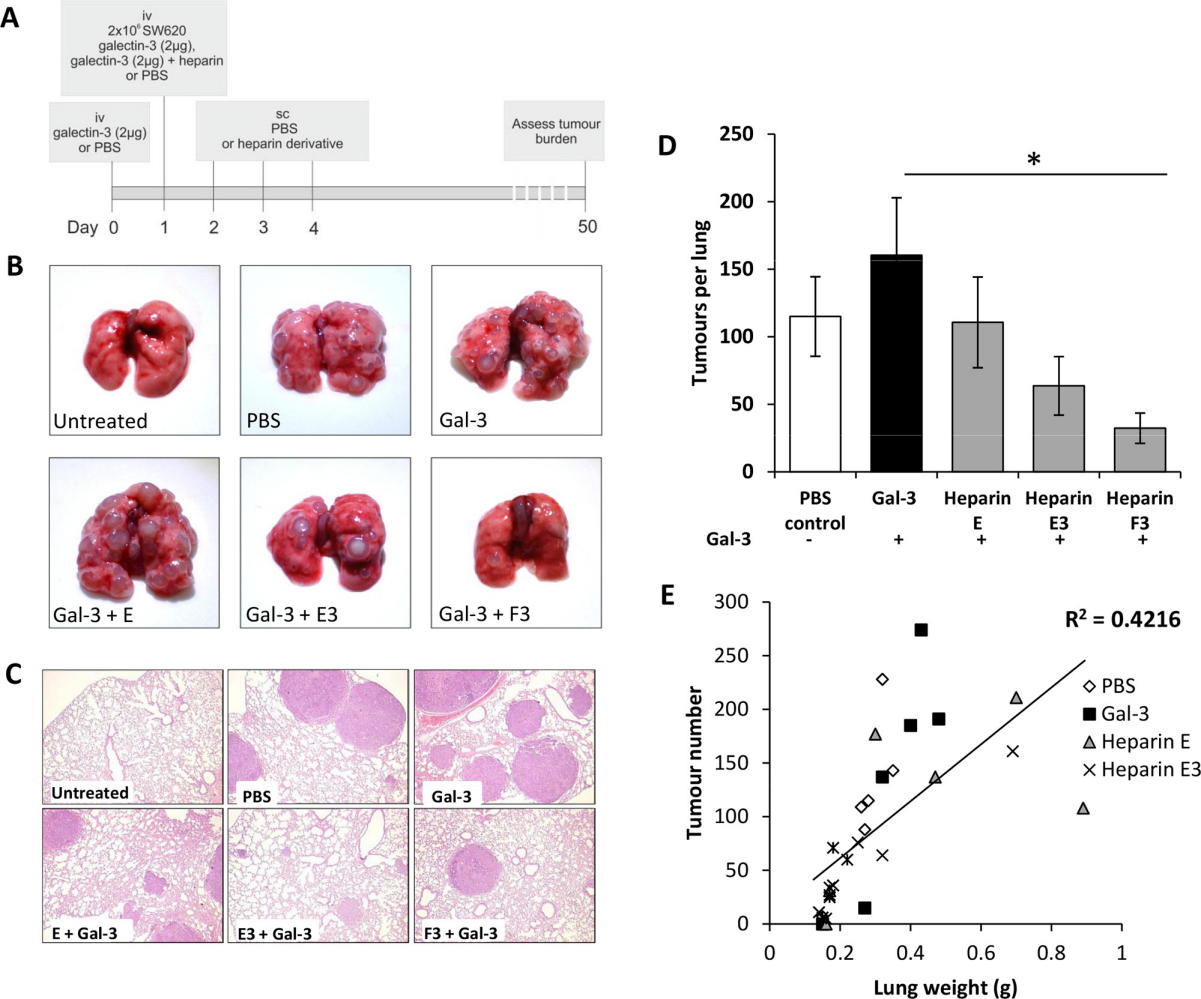
Heparin derivatives prevent galectin-3 mediated metastasis of human colon carcinoma SW620 cells in nude mice Schematic representation of experimental protocol **A.** Gross images of lungs **B.** or H and E stained photomicrographs **C.** from Balb/c nude mice administered with 2 × 10^6^ SW620 colon carcinoma cells iv and also co-injected with galectin-3 with or without heparin derivatives E, E3, F, F3, G or G3 (20 mg/kg) iv. Mean tumors per lung **D.** and lung weight vs tumor number **E.** are shown for all experimental groups. *p < 0.05 by one-way ANOVA, *n* = 6 mice per experimental group.

Similar effects were observed with human colon cancer SW620 cells in this mouse model. Approximately 40% increase in the number of metastatic foci per lung was observed in mice co-injected with a single tail vein injection of 2 μg galectin-3 in comparison to control mice after 7 weeks (Fig. 4A). Again, administration of the heparin derivatives E, E3 or F3 along with galectin-3 caused a reduction of metastatic foci per lung in comparison to the galectin-3-treated animals (Fig. 4B–4D; *p* < 0.05). A positive correlation of lung weight versus tumor number was observed across all treatment groups (Fig. 4E). Again, heparin F3 treatment resulted in a greater reduction in lung weight compared with all other groups and there were no significant differences in animal body weights among the animal groups during the experimental period (Supplementary Fig. S4B).

**Figure 5:**
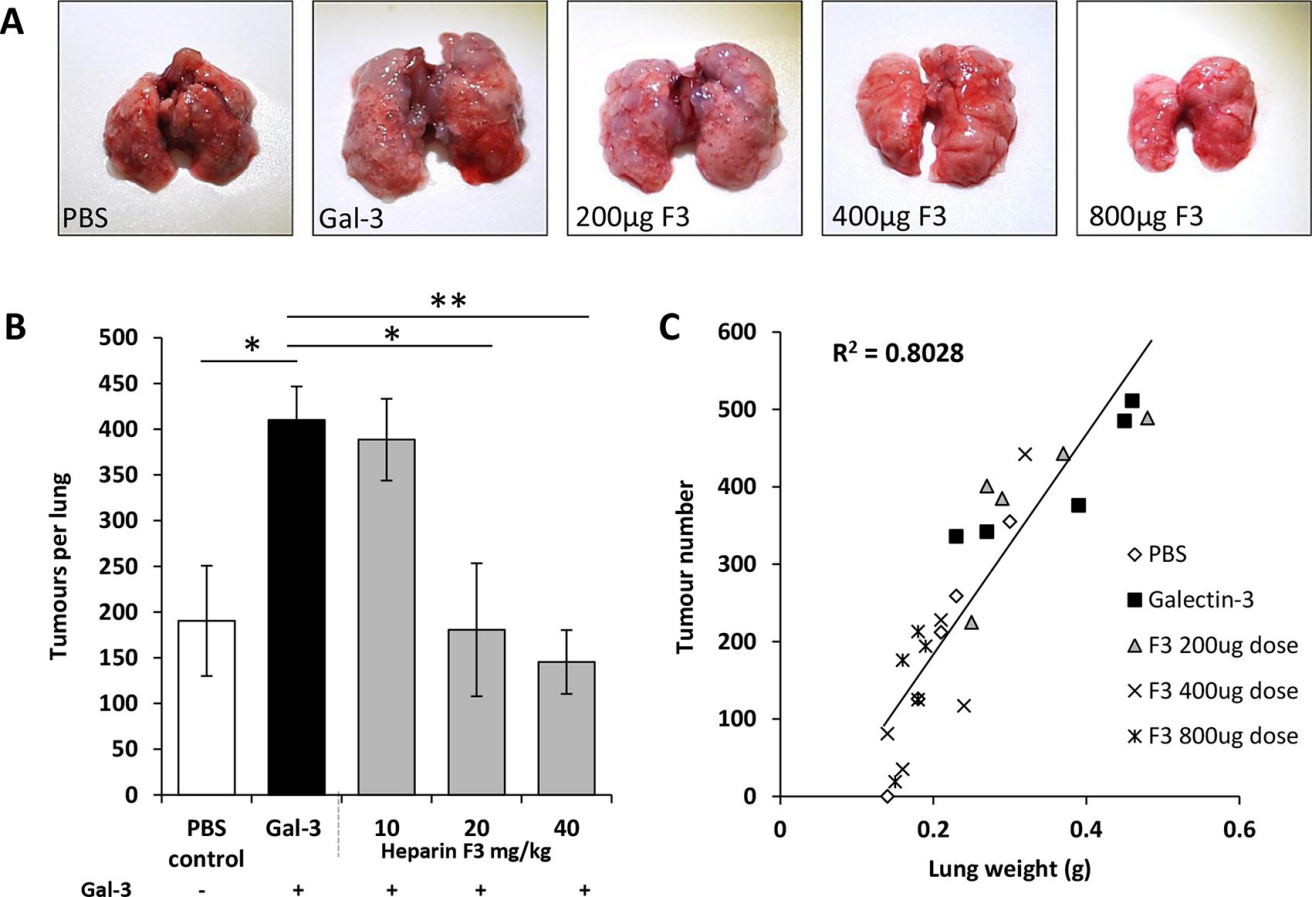
Dose-dependent inhibition of ACA19+ experimental metastasis by derivative F3 Gross images of lungs from Balb/c nude mice given galectin-3 alone or in combination with 10, 20 or 40 mg/kg heparin derivative F3 **A.** Mean tumors per lung **B.** and lung weight vs tumor number **C.** are shown for all experimental groups. *p < 0.05 by one-way ANOVA, *n* = 4 mice per experimental group.

To further assess the influence of these heparin derivatives on inhibition of galectin-3-mediated metastasis, three different doses (10, 20 or 40 mg/kg) of compound F3 were tested using the same dosing regimen as outlined in Fig. 3A. Again, a significant increase in number of lung metastatic foci occurred in mice treated with galectin-3 in comparison to the control group. Administration of either 20 mg/kg or 40 mg/kg, but not 10 mg/kg, of F3, caused a significant reduction in the number of metastatic nodules (Fig. 5A and 5B). A strong positive correlation was again observed between the tumor number and lung weight across all treatment groups (R^2^=0.8; Fig. 5C). No adverse effects or evidence of toxicity were observed in these mice following any dose or at any time-point. Together, these *in vivo* results indicate that these chemically-modified heparin derivatives inhibit circulating galectin-3-mediated metastasis and are well tolerated.

### Low sulfated heparin derivatives inhibit galectin-3-induced endothelial tubule formation

**Figure 6:**
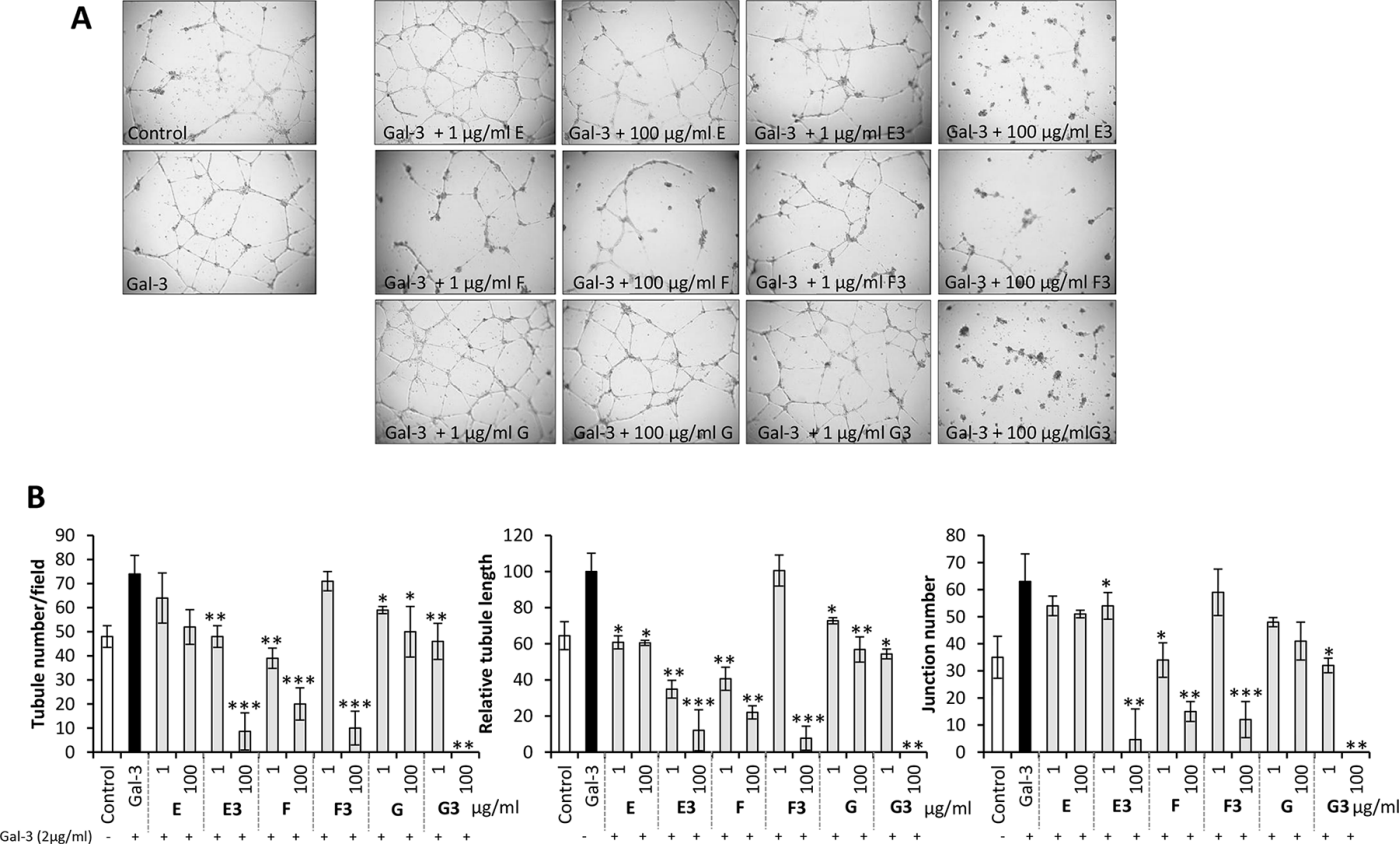
Modified heparin derivatives inhibit galectin-3-mediated endothelial tubule formation in angiogenesis Photomicrographs showing the effects of compounds E, E3, F, F3, G and G3 on galectin-3 mediated HUVEC tubule formation **A.** Quantification of tubule number, tubule length and junction number of HUVEC vessels following treatment with galectin-3 with or without 1 or 100 μg/ml heparin derivative **B.**

Increased tumor angiogenesis is another common effect of galectin-3 on cancer progression and metastasis [15, 16, 33, 34], and some modified heparins have previously been shown to have anti-angiogenic properties [35]. The effects of the heparin derivatives and their low molecular weight sub-fractions were therefore assessed on galectin-3-mediated angiogenesis *in vitro*. A 54.2 ± 16.0% increase in HUVEC tubule number, 55.1 ± 15.8% increase in tubule length and 80 ± 21.7% increase in junction number were observed in the presence of recombinant galectin-3 at 2 μg/ml (Fig. 6). Pre-incubation of galectin-3 with 1 or 100 μg/ml of each heparin derivative inhibited exogenous galectin-3-induced increase in endothelial tubule formation; all of the derivatives significantly reduced and in most cases completely abolished the galectin-3 induction in a dose dependent manner (Fig. 6). Of note, compound F and some of the ultra-low molecular weight sub-fractions (E3, F3, and G3) were able to inhibit tubule formation beyond control levels, indicating that these compounds are also able to inhibit angiogenesis beyond that induced by exogenous/circulating galectin-3 (Fig. 6). Indeed, when these compounds were tested in an *in vivo* chick chorioallantoic membrane angiogenesis model, compounds E and F exhibited significant inhibitory effects on VEGF-induced angiogenesis, particularly in the case of F which exerted > 95% inhibition (Supplementary Fig. S5; *p* < 0.05).

### Low sulfated heparin derivatives inhibit cancer-endothelial cell adhesion mediated by cancer cell-associated galectin-3

**Figure 7:**
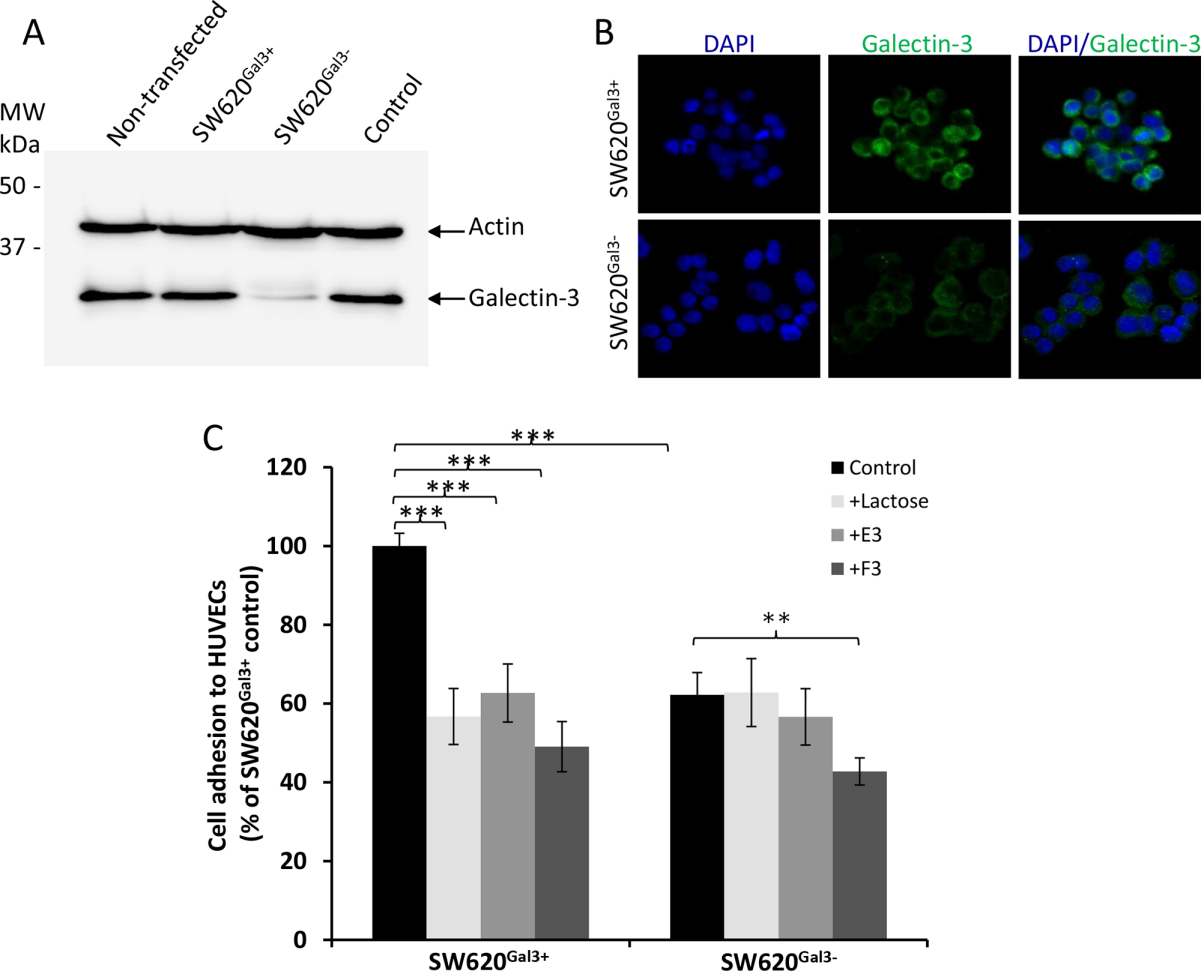
Modified heparin derivatives inhibit endogenous galetin-3-mediated cancer cell-endothelial adhesion shRNA galectin-3 knockdown in SW620 cells (A and B). Dual immunoblotting of the transfected cells with antibodies against galectin-3 and actin shows 82% reduction of galectin-3 expression (SW620^Gal3−^) in comparison to non-transfected, control or negatively selected (SW620^Gal3+^) cells **A.** Galectin-3 immunohistochemistry shows marked reduction of galectin-3 expression in galectin-3 knockdown SW620^Gal3−^ cells in comparison to SW620^Gal3+^ **B.** E3, F3 or lactose inhibit adhesion to HUVEC of SW620^Gal3+^ but not (or much less than) SW620^Gal3−^cells **C.** Adhesion of the transfected cells to HUVEC monolayer was conducted in the presence of 100 ug/ml lactose, E3 or F3. The data are expressed as mean ± SEM of three independent experiments, each in triplicate, ***p < 0.001, **p < 0.01, ANOVA.

To assess whether these modified heparin derivatives also affect endogenous galectin-3-mediated activities, we suppressed galectin-3 expression in SW620 cells using shRNA. The stably transfected cells showed 84% reduction of galectin-3 expression in comparison to the un-transfected or negatively-transfected cells (Fig. 7A). Suppression of galectin-3 expression was seen to be associated with a 38% reduction in the adhesion of these cells to HUVEC cells when compared with the galectin-3-expressing cells (Fig. 7B), confirming a role of cancer cell-associated galectin-3 in cancer cell-endothelial adhesion, as shown by several previous studies [36, 37]. Whilst lactose and the modified heparin derivative E3 both caused significant inhibition (43 and 37%, respectively) of cell adhesion to HUVEC of the galectin-3-expressing SW620^Gal3+^ cells, neither of them showed an effect on the adhesion of galectin-3-suppressed SW620^Gal3−^ cells. Also, the inhibitory effect (51%) of heparin derivative F3 on SW620^Gal3+^ adhesion to HUVEC was substantially reduced in SW620^Gal3−^ cells (32%) (Fig. 7B). Together, these results indicate that the heparin derivatives also inhibit cell adhesion mediated by cancer cell-associated galectin-3. This is in keeping with the observed further inhibition of lung metastasis below the numbers seen in the control animals that had not received exogenous galectin-3 (Fig. 4D and 5D), and supports an inhibitory effect of the heparin derivatives also on endogenous galectin-3-mediated actions in metastasis.

### NMR analysis of galectin-3 interaction with the heparin derivatives

**Figure 8:**
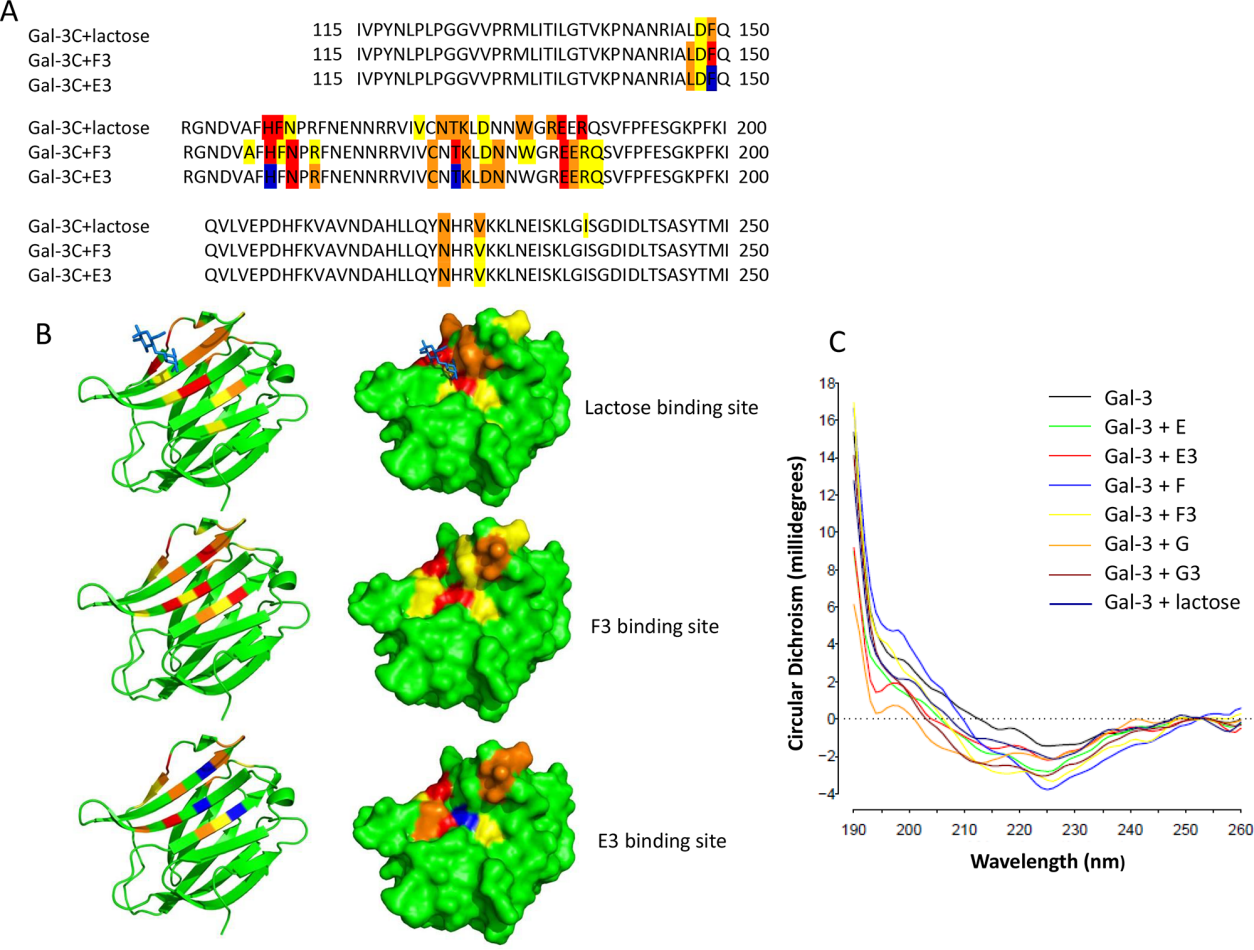
The modified heparin derivatives bind to the galectin-3 carbohydrate-recognition domain and induce galectin-3 conformation changes NMR analysis reveals the binding sites of lactose and the heparin derivatives on galectin-3 CRD **A.** Residues highlighted in red show the most significant chemical shift changes, followed by orange and yellow. Residues highlighted in blue correspond to signals absent from the Gal-3C with E3 spectrum. Surface and cartoon representations show lactose, F3 and E3 binding sites on the galectin-3 CRD **B.** Synchrotron radiation circular dichroism (SRCD) spectroscopy analysis shows galectin-3 conformation changes upon interaction with lactose and the modified heparin derivatives **C.** Different structural changes of galectin-3 are shown upon interaction with lactose and the heparin derivatives E, E3, F, F3, G and G3.

To gain insights into the molecular interaction of these heparin derivatives with galectin-3, we employed NMR (nuclear magnetic resonance). The 2D ^1^H-^15^N HSQC spectra of the protein in the absence and presence of the ligands were compared; residues whose chemical shifts were affected by the presence of the ligands were deemed to be either directly involved with binding the ligands or indirectly affected due to conformational/environmental changes. This method allows the identification of the ligand binding sites. In the presence of lactose, resonances from His158, Asn160, Glu184, Asn174 and Trp181 (Supplementary Fig. S6 and Fig. 8A and 8B) showed significant chemical shift changes. These data are in agreement with the previously published crystallography structure of the Gal-3C-lactose complex [38] and indicate the critical involvement of these amino acids in lactose binding. Some of these amino acids (e.g. His158 and Asn160) were also seen to be involved in F3 and E3 binding. In addition, the resonance of some residues such as Phe 149, Asn160 and Thr175, which were not perturbed by lactose binding, appeared to be affected by F3 binding. Resonances from Phe149 and Thr 175, but not Asn 160, appeared to be perturbed by E3 binding. The binding of the modified heparin derivatives to galectin-3 at 1:20 titration induced a similar magnitude of chemical shift changes to lactose, suggesting that the binding affinities of lactose, E3 and F3 are probably similar (lactose has been reported to bind galectin-3 with a Kd of 110 μM, [16]. These results indicate that the heparin derivatives bind to the galectin-3 CRD domain in a similar manner as lactose, although slightly more amino acids appear to be involved with the E3 and F3 binding; this is not surprising given that these heparin derivatives are larger than lactose and hence able to form a larger carbohydrate:protein interface.

We also assessed the interaction of these heparin derivatives with galectin-3 in solution by Synchrotron Radiation Circular Dichroism (SRCD) spectroscopy. SRCD spectroscopy conducted in the far-UV region (125–260 nm) is highly sensitive to changes in protein secondary structure [30] in solution and provides unequivocal evidence of conformational change in a protein upon ligand binding. It was found that the presence of fractions E, F and G and their respective sub-fractions E3, F3 and G3, like lactose, all caused distinct secondary structural changes in galectin-3. These results are consistent with the NMR finding. Interestingly, each fraction induced slightly different secondary structural changes in galectin-3, implying distinctive modes of interaction (Fig. 8C).

## DISCUSSION

This study shows that several chemically modified, de-*O*-sulfated, *N*-acetylated heparin derivatives are effective galectin-3 binding inhibitors. Interaction of these low sulfated heparin derivatives with galectin-3 alter galectin-3 conformation and inhibit galectin-3-ligand binding, galectin-3-mediated cancer cell-endothelium adhesion and galectin-3-mediated angiogenesis *in vitro* and abolish circulating galectin-3-mediated increase of metastasis *in vivo* in a mouse model of cancer metastasis. Moreover, these modified heparin derivatives show no anticoagulant activity, no effect on E-, L- or P-selectin binding to sialyl Lewis^X^ antigen and no detectable cytotoxicity.

Galectin-3 is overexpressed by most types of cancer cells and is involved in cancer development, progression and metastasis. Galectin-3 is found intracellularly, on the cell surface and also in the circulation. Cytoplasmic galectin-3 is an apoptosis inhibitor and promotes neoplastic transformation and tumorigenesis [39]. Galectin-3-expressed on the surface of cancer cells acts as an adhesion molecule and promotes cancer cell-cell and cell-matrix interaction during cancer cell invasion and migration [40]. Extracellular galectin-3 binds to cell surface N-glycans and induces monocyte and T cell apoptosis thus facilitating tumor cell evasion from immune surveillance [40, 41] whilst circulating galectin-3 promotes disseminating tumor cell adhesion to vascular endothelium and increases the formation of tumor microemboli thus prolonging tumor cell survival in the circulation [10]. Such broad cancer- and metastasis-promoting actions of galectin-3 have prompted many laboratories over recent years to explore therapeutic strategies that target galectin-3. These include the development of a TF antigen-mimicking vaccine, anti-MUC1 antibodies, truncated galectin-3 form [42], peptide-based inhibitors [43], carbohydrate-based small synthetic inhibitors [44–47] and natural galectin-3 inhibitors such as modified citrus pectin [48]. Lactose and galactose are themselves natural inhibitors of galectin-3, though they have weak binding affinity [10, 11, 18].

Previous studies have shown that standard, unfractionated heparins have a variety of anti-metastatic activities that include inhibition of tumor growth, inhibition of selectin-mediated tumor cell interaction with leukocytes and endothelial cells, inhibition of angiogenesis, growth factor-mediated processes and the inhibition of tumor cell emboli formation [49]. Unfractionated heparin blocks binding of P- and L-selectins and thus prevents tumor cells from forming micro-emboli through interaction with platelets and leukocytes. However, blocking the action of selectins may compromise immune responses by impairing recruitment of leukocytes. A non-anticoagulant N-acetylated heparin reduces metastasis in P- and L-selectin deficient mice [50] suggesting that selectin inhibition is not the only mechanism by which heparin exerts its anti-tumor effects. The low-sulfated heparin derivatives identified in this study, which do not affect binding of selectins, may be better suited to therapeutic anti-cancer use.

It is interesting that these modified heparin derivatives, although not galactose-containing and thus lacking the canonical binding moiety for this class of lectins, can inhibit galectin-3 binding and galectin-3-mediated cell-cell interactions. The galectin-3 carbohydrate recognition domain (CRD), which includes 127 amino acid residues, contains several stranded β-sheets which form a binding pocket. More than 10 of the CRD amino acid residues are known to be involved in galectin-3 binding to TF disaccharide as revealed by NMR analysis [51]. Our NMR binding analysis showed that the heparin derivatives F3 and E3 bind to the galectin-3 CRD domain, associating with similar residues as utilised by lactose, implying binding to the same site. The small variations in chemical shift changes between F3, E3 and lactose were to be expected given the differences in the ligand sizes. In addition, circular dichroism data showed that F3 and E3 binding, like lactose, also induced conformational changes in galectin-3 (Fig. 8). Previous studies have shown that several 15∼16 mer synthetic peptides identified by bacteriophage display technology are strong galectin-3 binding inhibitors (kd 17–80 nM) [43]. These synthetic peptides, although not modified with any galactose residues, were reported to bind to the galectin-3 CRD and inhibit galectin-3-TF interaction, and prevent galectin-3-mediated cancer cell adhesion, aggregation *in vitro* and metastasis *in vivo* [52]. Thus non-galactose-containing molecules are capable of being galectin-3 inhibitors if they possess appropriate binding properties and conformations.

It should be mentioned that our previous studies have reported that endotoxin content in recombinant galectin-3 is negligible and does not affect galectin-3 activity [9]. It is therefore extremely unlikely that endotoxin will have any influence on the effect of heparin derivatives shown in this study. Moreover the galectin-3 effects, that were inhibitable by some of the modified heparins, were themselves inhibitable by TF-expressing glycans/lactose indicating that they were not related to any endotoxin contamination.

It was noticed that administration of the low molecular weight compounds E3 and F3 not only abolished the lung metastasis induced by exogenously introduced galectin-3, but also produced further inhibition of lung metastasis below the numbers seen in the control animals, that had not received exogenous galectin-3. This implies additional anti-metastatic effects of the compounds that are independent of interaction with exogenous galectin-3. Endogenous galectin-3 expressed on the surface of cancer cells [40] [both SW620 [53]and ACA19+ [54] cells express galectin-3 and vascular endothelial cells [55] is also known to enhance metastasis by acting as an adhesion molecule to promote cancer-endothelial cell adhesion. The discovery that suppression of galectin-3 expression in SW620 cells by shRNA abolished or largely reduced the inhibitory effect of these heparin derivatives on adhesion of the cells to endothelial cells (Fig. 7) supports this. The importance of galectin-3 in promoting angiogenesis has been reported previously [15, 16] so the anti-angiogenic action of these compounds may also contribute to these additional anti-metastasis effects.

It is also noteworthy that the Balb/c nude mice used in this study have no T cells. As galectin-3 can also act on tumor-infiltrating T cells to impair their anti-tumor function [41], it is possible that these chemically-modified heparin derivatives could possess additional anti-tumor effects in cancer patients as a result of inhibition of galectin-3-mediated T cell apoptosis.

In summary, several chemically modified, low sulfated heparin derivatives have been identified as potent galectin-3 binding inhibitors. These heparin derivatives bind to the galetin-3 carbohydrate-recognition domain and inhibit galectin-3-ligand binding and galectin-3-mediated metastasis. Importantly, these chemically modified heparin derivatives have no anti-coagulant activities, no effect on P-, L- or E-selectin-ligand binding and no detectable cytotoxicity. They therefore represent a very promising new class of therapeutic agents to target galectin-3-mediated metastasis.

## MATERIALS AND METHODS

### Production of chemically modified heparin derivatives

Standard heparin (average MW∼12–15 kDa) was chemically modified by selective desulfation as previously described [25]. Structural modifications were confirmed by NMR spectroscopy (Supplementary Tables S1 and S2). Different size fractions were produced from these modified heparins by partial depolymerisation using chemical β-elimination [26]. Three broad size fractions were then separated by subjecting the partially depolymerised materials to gel filtration chromatography using Sephadex G-100 (Supplementary Fig. S1). The resulting size fractions were then assessed as ultra-low molecular weight (<3 kDa), low molecular weight (∼3–7 kDa) and intermediate molecular weight (>7 kDa), using heparin fractions of known sizes as reference standards.

### ELISA

Ninety-six well plates were coated overnight with coating buffer (15 mM Na_2_CO_3_, 17 mM HaHCO_3_, pH 9.6) containing asialo-fetuin (ASF; 20 μg/ml) or bovine serum albumin (BSA; 20 μg/ml). Asialo-fetuin (sigma), produced by neuraminidase treatment of fetuin that removes terminal sialic acid and exposes galactose residues, contains several terminal galactose residues [27] and is a typical galectin-3 binding ligand and is recognized by other galectin members such as galectin-1, -2, -4 and -8 (2). Galectin-3 (2 μg/ml), heparin derivatives (0–200 μg/ml) or lactose (100 μM) were added to a second BSA-coated 96-well plate for 15 minutes prior to transfer onto the assay plate for 1 hour at room temperature. Plates were washed with PBS and probed with a biotin-conjugated anti-galectin-3 antibody (0.5 μg/ml; R&D Systems BAF1154) for 1 hour and detection was carried out using ExtrAvidin-peroxidase and OPD (Sigma) as per the manufacturer's instructions. OD values were determined at 492 nm with a reference at 595 nm using a micro plate reader (Tecan, Männedorf, Switzerland).

### Synchrotron radiation circular dichroism (SRCD) spectroscopy

SRCD was carried out using Diamond B23 beamline Module B end-station with calcium fluoride cells of path length 0.1mm (190–260 nm). Samples that were assessed were galectin-3 (0.5 mg/ml), alone and galectin-3 mixed with each of the six heparin derivatives (0.5 mg/ml) in PBS. The SRCD analysis reports on structural changes within the galectin-3 molecule upon interaction with each heparin derivative in solution. The spectral analyses do not describe the degree of galectin-3 inhibition but determine whether each heparin derivative causes the same or different changes in galectin-3 conformation.

### Production of ^15^N-labelled recombinant galectin-3

The cDNA sequence encoding the C-terminal carbohydrate recognition domain (CRD) of galectin-3 (Gal-3C) (residues 115–250) was cloned into a pETM11A expression vector. The vector was transformed into *E.coli* BL21(DE3) strain by addition of 50 ng of the plasmid to 50 μl of BL21 cells, followed by heat shock for 45 seconds at 42°C then 1 hr recovery in LB media at 37°C. Transformants were selected with 32 μg/ml kanamycin and grown in minimal medium with ^15^NH_4_Cl as the nitrogen source. The protein expression was induced with 1 mM IPTG when OD600 reached 0.6–0.85. Following overnight incubation at 18°C, the cells were harvested and lysed using a cell homogenizer. The cell supernatant was applied to a HisTrap FF 5 ml column (GE Healthcare) and the His-tagged recombinant Gal-3C was eluted with 500 mM Imidazole. The eluted protein was treated with TEV protease (1 mg of TEV protease/15 mg of the protein) to remove the His-tag. After application onto the HisTrap FF 5 ml column to remove the cleaved His-tag and TEV protease, the proteins were further purified using a Superdex75 16/60 column (GE Healthcare), the purified recombinant Gal-3C was eluted at 220 and 260 ml. SDS-PAGE analysis revealed the purity of the recombinant Gal-3C to be >95%.

### NMR analysis

Galectin-3 interactions with lactose and the heparin derivatives E3 and F3 were analysed by NMR at 298 K in a Bruker 800 MHz solution-state spectrometer equipped with cryogenic probe head. 2D ^1^H-^15^N HSQC experiments were performed on ^15^N-labelled Gal-3C at a concentration of 100 μM in 50 mM Phosphate buffer, 100 mM NaCl pH6.7. 2D ^1^H-^15^N HSQC spectra were acquired at 1:1, 1:2.5, 1:5, 1:10 and 1:20 protein:ligand ratios. Data were processed using the Bruker Software TopSpin and analysed using CCPN software [28]. Resonance assignments were transferred from Biological Magnetic Resonance Bank (BMRB) Accession No 4909, with some additional assignments made within this work. The ligand binding sites were mapped using chemical shift changes between Gal-3C and Gal-3C-ligand spectra [29, 30].

### Cell lines

ACA19+ cells, previously stably transfected with human *MUC1* of human melanoma A375 cells [31], were a gift from Dr John Hilkens (The Netherland Cancer Institute) in 2012. The human SW620 colorectal adenocarcinoma cells obtained in 1998 from the European Cell Culture Collection (Wiltshire, UK) express high levels of cell surface MUC1 [32]. The cell lines were last authenticated by DNA profiling (DNA Diagnostics Center, London; May 2014). ACA19+ and SW620 cells were maintained in Dulbecco's Modified Eagle's medium containing 10% fetal calf serum. Human umbilical vein endothelial cells (HUVEC) cells were maintained in EBM-2 culture medium (EGM-2 BulletKit; Lonza).

### Generation of stable galectin-3 knockdown cells

SW620 cells were seeded at 0.5 × 10^5^ cells/ml in 96-well plates and incubated at 37°C until 50–60% confluent. Cells were then treated with 100 ng MISSION shRNA plasmid DNA (TRCN0000029305, Sigma) or empty vector control (SHC002, Sigma) with the transfection reagent metafectene (Biontex) in a 1:4 plasmid:metafectene (w/v) ratio for 6 hr at 37°C. The transfected cells were selected by treatment with 8 μg/ml puromycin over 72 hr, followed by 48 hr with 4 μg/ml puromycin. Cells were then released by trypsin, suspended in complete culture medium to highly-diluted cell suspension and plated in a 96-well plate. Wells containing a single cell were identified by microscope. Following further culture to allow cell proliferation and colony formation, galectin-3 expression was determined by galectin-3 immunoblotting and immunohistochemistry to allow separate selection of galectin-3 expressing (SW620^Gal3+^) and knockdown (SW620^Gal3−^) cells/colonies.

### Galectin-3 immunofluorescence

The transfected cells were seeded at 5 × 10^4^ cells/well into 24-well plates with glass coverslips inserted and cultured until 50–70% confluent. Cells were washed with PBS, fixed with 2% paraformaldehyde and treated with blocking solution (1% BSA/PBS) for 45 min before application of 2 μg/ml anti-human galectin-3 antibody (R&D Systems) for 2 hr. After two washes with PBS, FITC-conjugated secondary antibody (1:1000 dilution) was applied for 1 hr. After 6 washes with PBS, the cells were mounted using DAPI-containing fluorescent mounting medium (Vector) and images were acquired using an Olympus BX51 fluorescent microscope.

### Adhesion assay

HUVEC cells were plated into 96-well plates at 10^4^ cells/well and cultured for two days at 37°C. ACA19+, SW620^Gal3+^ and SW620^Gal3−^ cells were harvested using non-enzymatic cell dissociation solution (Sigma) and were incubated with Calcein AM (Invitrogen) at 37°C in the dark for 30 minutes. HUVEC monolayers were washed twice with PBS. Galectin-3 (final concentration 1 μg/ml) and heparin derivatives (final concentration 0.1–100 μg/ml) were added to HUVEC monolayers in 50 μl serum-free media (containing 0.5 mg/ml BSA). The cells were washed twice with PBS and 50 μl of 2 × 10^5^ cells/ml were added to each well. Cells were allowed to adhere for 1 hr and plates were washed gently twice. Adherent cells were lysed with 0.25% SDS and the fluorescent intensity was measured by a fluorescence plate reader at excitation 485 nm and emission 535 nm.

### Angiogenesis assay

Fifty microlitres per well of 10 mg/ml matrigel (BD Biosciences) was plated in 96-well plates and allowed to set in an incubator at 37°C. HUVEC cells were plated at 1.5 × 10^4^ cells/well in reduced growth factor EGM-2 media (50% kit concentration) containing galectin-3 (2 μg/ml) without or with heparin derivative (1–100 μg/ml) or PBS for 24 hr. The tubule number, tubule length and junction number were then quantified from one low power field per well. Tubule length was quantified using ImageJ software.

### Anticoagulant activity measurements

For Factor Xa and Factor IIa assays, increasing concentrations of heparin or heparin derivatives (in 0.9% NaCl) were incubated for 2 minutes at 37°C with 2 nKat/ml bovine Factor Xa or bovine Factor IIa (both from Sigma-Aldrich, in dH2O) and 0.003 IU/ml bovine antithrombin III (Sigma-Aldrich, in 50 mM Tris HCl, 0.175 M NaCl and 7.5 mM EDTA). Two hundred μM Factor Xa chromogenic substrate (Sigma-Aldrich, in dH2O) was added and samples were incubated for another 45 seconds at 37°C. The reaction was stopped with 25% glacial acetic acid. Colour change was assessed by absorbance at 405 nm.

Activated Partial Thromboplastin Time (aPTT Assay) was carried out in a cylindrical cuvette (Behnk Elektronik). The cuvette was pre-warmed to 37°C on a heating block, placed in the coagulation analyser (Axis Shield Thrombotrack) and charged with a ball bearing, normal citrated plasma (50 μL, Technoclone), Pathromtin SL (50 μL, Siemens) and test sample (25 μL). Following incubation for 2 minutes, pre-warmed (37°C) calcium chloride (25 μL) was added and the coagulation analyser was initiated. Clotting times are reported as the time at which the coagulometer was no longer able to stir the sample. Unclotted samples after 120 seconds were recorded as ‘no clot.’

Prothrombin Time (PT Assay) was carried out in a cylindrical cuvette (Behnk Elektronik). The cuvette was pre-warmed to 37°C on a heating block, placed in the coagulation analyser (Axis Shield Thrombotrack) and charged with a ball bearing and normal citrated plasma (50 μL, Technoclone). Following incubation for 1 minute, pre-warmed (37°C) test sample (50 μL) was added along with Thromborel^®^ S reagent (50 μL, Siemens). Upon addition of the final reagent, the coagulation analyser was initiated. Clotting times are reported as the time at which the coagulometer was no longer able to stir the sample. Unclotted samples after 120 seconds were recorded as ‘no clot.’

### Selectin binding analysis

Recombinant human L-selectin (10 ng/ml), P-selectin (10 ng/ml) or E-selectin (30 ng/ml) Fc chimeras (R&D Systems) were mixed with increasing concentrations of heparin or heparin derivatives in PBS before addition to 96-well ELISA plates precoated with BSA-Sialyl Lewis^x^ (110 ng/well, R&D Systems) for 45 minutes at room temperature. The plates were washed three times with 200 μl of Quantikine wash buffer (R&D Systems) and the bound selectins were detected using an HRP-anti Fc antibody and Substrate Reagent Pack (R&D Systems) according to the manufacturer's instructions.

### Cytotoxicity assay

HUVEC, ACA19+ or SW620 cells were plated at 5000 cells/well in 100 μl of EBS+ medium (Lonza; for HUVEC cells) or DMEM supplemented with 10% FBS (for ACA19+ and SW620 cells). Cells were incubated with 100 μg/ml of derivatives for 24 or 48 hr. Cell death was measured using an LDH-Cytotoxicity Assay Kit II (Abcam) as per the manufacturer's instructions.

### *In vivo* metastasis

Female Balb/c nude (CAnN.Cg-FOX1NU/Crl) mice aged 6–7 weeks were purchased from Charles River Laboratories (Margate, UK) and maintained at the University of Liverpool in specific pathogen-free conditions with a 12:12 hour light:dark cycle. All animal studies were conducted with UK Home Office and local ethics committee approval from the University of Liverpool.

Mice were administered PBS, galectin-3 (2 μg) or galectin-3 plus 20 mg/kg heparin derivative in PBS, via tail vein injection. Twenty four hr later, a second intravenous injection was conducted with either 1 × 10^6^ ACA19+ cells/mouse or 2 × 10^6^ SW620 cells/mouse that had been pre-incubated with PBS, galectin-3 (2 μg) or galectin-3 plus heparin derivative (20 mg/kg) for 1 hour prior to injection. Subcutaneous injections were then administered daily for 3 days with either PBS or heparin derivative (20 mg/kg). Mice were sacrificed by cervical dislocation 5 (ACA19+) or 7 (SW620) weeks following the tumor cell injection and organs were harvested for macroscopic and microscopic assessment. Tumor number on the surface of the lungs was assessed using a dissection microscope (Motic, Redding, UK). Lung weight was also assessed as an indicator of tumor burden.

Tissue was fixed overnight in 4% formalin, processed and paraffin-embedded. Sections of organs (lungs, brain, kidney, liver, small intestine, colon, stomach, spleen) were assessed for tumor formation from H and E sections. Lung tumor size was quantified using a graticule and x5 objective on a light microscope. All tumors on one section per lung were assessed for their maximum dimension (the majority were spherical nodules).

### Statistical analysis

All data were found to be normally distributed and were assessed by one-way ANOVA. Statistical significance was reported if *p* < 0.05.

## SUPPLEMENTARY MATERIALS



## ABBREVIATIONS

APTT: activated partial thromboplastin time
CRD: carbohydrate recognition domain
HUVECs: human umbilical vein endothelial cells
LMWH: low molecular weight heparins
NMR: nuclear magnetic resonance
PT: prothrombin time
TF: Thomsen Friedenreich (Galβ13GalNAc-) antigen
UFH: unfractionated heparin
