# Supplementary material for: Chemically modified, non-anticoagulant heparin derivatives are potent galectin-3 binding inhibitors and inhibit circulating galectin-3-promoted metastasis
Source: Oncotarget. 2015 Jun 23;6(27):23671–87. doi: 10.18632/oncotarget.4409 (PMC4695144; doi:10.18632/oncotarget.4409)
Supplement: Supplementary file 1 [file oncotarget-06-23671-s001.pdf]

SUPPLEMENTARY MATERIALS

Supplementary Table S1. Disaccharide compositions of the chemically-modified heparin derivatives*

	Disaccharide standard								
	D1 ΔUA-GlcNAc	D2 ΔUA-GlcNAc(6S)	D3 ΔUA-GlcNS	D4 ΔUA-GlcNS(6S)	D5 ΔUA(2S)-GlcNS	D6 ΔUA(2S)-GlcNS(6S)	D7 ΔUA(2S)-GlcNAc	D8 ΔUA(2S)-GlcNAc(6S)	%
**A**	6.8	-	3.4	13.4	7.0	67.4	-	2.0	100
**B**	14.27	7	-	-	-	3.5	-	75.3	100
**C**	7.1	-	13.7	79.2	-	-	0	0	100
**D**	15.5	-	43.2	7	34.3	-	-	-	100
**E**	14.4	62.9	3.4	19.3	-	-	-	-	100
**F**	55.8	7.7	-	-	-	-	34.8	1.7	100
**G**	19.1	-	79.1	-	-	1.8	-	-	100
**H**	99	-	-	-	-	-	-	1.0	100

*The modified heparin derivatives were exhaustively digested with a mixture of heparitinases I, II and III to their constituent disaccharides (here denoted D1 to D8). The resulting component disaccharides were separated by strong-anion exchange HPLC (Propac PA-1 column, Dionex UK) and quantified (A232) with reference to authentic standards [1].

Supplementary Table S2. ^{1}H and ^{13}C NMR chemical shift values for the chemically-modified heparin derivatives*

	Glucosamine						Iduronate				
**Polysaccharide**	**A-1**	**A-2**	**A-3**	**A-4**	**A-5**	**A-6**	**I-1**	**I-2**	**I-3**	**I-4**	**I-5**
A	99.5	60.7	72.5	78.8	72.0	69.2	102.1	78.9	72.1	79.0	72.3
	5.42	3.31	3.69	3.79	4.05	4.30–4.42	5.23	4.37	4.22	4.14	4.82
B	96.6	56.2	73.0	79.3	72.3	69.6	102.2	76.8	67.3	74.2	70.8
	5.15	4.03	3.76	3.78	4.04	4.31–4.37	5.20	4.37	4.31	4.08	4.91
C	98.1	60.3	72.4	80.1	71.5	68.7	104.6	71.1	70.4	77.2	71.2
	5.34	3.24	3.65	3.71	4.02	4.36–4.23	5.04	3.78	4.12	4.08	4.84
D	100.0	60.8	72.4	80.5	73.8	62.6	102.0	77.6	70.7	78.7	71.4
	5.31	3.27	3.71	3.70	3.89	3.86–3.88	5.26	4.35	4.25	4.06	4.84
E	97.1	56.2	72.5	79.6	71.8	68.8	104.6	72.0	71.4	77.0	71.9
	5.18	4.00	3.78	3.79	4.08	4.37–4.26	5.01	3.75	3.42	4.10	4.78
F	96.8	56.6	72.9	80.6	74.2	62.9	102.3	76.6	67.1	74.1	70.6
	5.14	4.03	3.79	3.76	3.91	3.87–3.92	5.26	4.37	4.28	4.07	4.91
G	98.2	60.5	72.5	80.2	73.5	62.4	104.3	72.2	71.5	77.8	72.2
	5.39	3.26	3.67	3.72	3.87	3.84–3.88	4.95	3.74	4.11	4.08	4.77
H	97.1	56.2	72.3	79.6	73.7	62.3	104.3	72.5	72.2	77.3	72.6
	5.18	3.97	3.76	3.74	3.89	3.85–3.88	4.92	3.69	3.89	4.07	4.73

*The derivatives were characterised by ^{1}H and ^{13}C NMR to confirm their structure. NMR spectra were recorded in D_2O at 40°C on a 400 MHz instrument. Assignment was by a combination of COSY, TOCSY, HMBC two-dimensional spectra. ^{13}C spectra were recorded on 150 mg samples of the polysaccharide. Chemical shift values were recorded relative to trimethylsilyl propionate as reference standard at 40°C. The 1H chemical shift values quoted for position-6 of glucosamine residues (A-6) are intervals. Signals from the carbonyl group of iduronate and acetyl CH3 groups of N-acetylated glucosamine derivatives are not shown. The ^{1}H chemical shift values quoted for position-6 of glucosamine residues (A-6) are intervals. Signals from the carbonyl group of iduronate and acetyl CH_3 groups of *N*-acetylated glucosamine derivatives are not shown.

Supplementary Table S3. Chemically-modified heparin derivatives, sub-fractions and their structures

Modified derivatives		Structural Modification	Molecular weight (kDa)
**C**	2-de-O-sulfated	I2OH[a]	
**C1**		I2OH	>7000
**C2**		I2OH	7000–3000
**C3**		I2OH	<3000
**D**	6-de-O-sulfated	A6OH[b]	
**D1**		A6OH	>7000
**D2**		A6OH	7000–3000
**D3**		A6OH	<3000
**E**	2-de-O-sulfated, N-acetylated	I2OH, NAc	
**E1**		I2OH, NAc	>7000
**E2**		I2OH, NAc	7000–3000
**E3**		I2OH, NAc	<3000
**F**	6-de-O-sulfated, N-acetylated	A6OH, NAc	
**F1**		A6OH, NAc	>7000
**F2**		A6OH, NAc	7000–3000
**F3**		A6OH, NAc	<3000
**G**	2, 6-de-O-sulfated	I2OH, A6OH	
**G1**		I2OH, A6OH	>7000
**G2**		I2OH, A6OH	7000–3000
**G3**		I2OH, A6OH	<3000
**H**	2, 6-de-O-sulfated, N-acetylated	I2OH, A6OH, NAc	
**H1**		I2OH, A6OH, NAc	>7000
**H2**		I2OH, A6OH, NAc	7000–3000
**H3**		I2OH, A6OH, NAc	<3000

[a]I = Iduronic acid; [b]A= glucosamine

Supplementary Table S4. No detectable cytotoxic effect of the heparin derivatives on human cancer (ACA19+ and SW620) or endothelial (HUVEC) cells[*]

Compound(100ug/ml)	LDH (SW620 cells)	LDH (ACA19+ cells)	LDH (HUVEC cells)
**Heparin**	NDI[a]	NDI	NDI
**E**	NDI	NDI	NDI
**E3**	NDI	NDI	NDI
**F**	NDI	NDI	NDI
**F3**	NDI	NDI	NDI

[a]NDI: no detectable increase of cellular LDH release in comparison to the non-treated control cells after 48 hr compound exposure

[*]Sub-confluent SW620, ACA19+ or HUVEC cells in 96-well plates were incubated at 37°C for 48 hours with 100 µg/ml of heparin or heparin derives. The medium was removed and the amount of lactate dehydrogenase present was determined using a LDH-Cytotoxicity Assay Kit II. Cells treated overnight without or with 1 µM staurosporine were used as negative and positive controls respectfully.

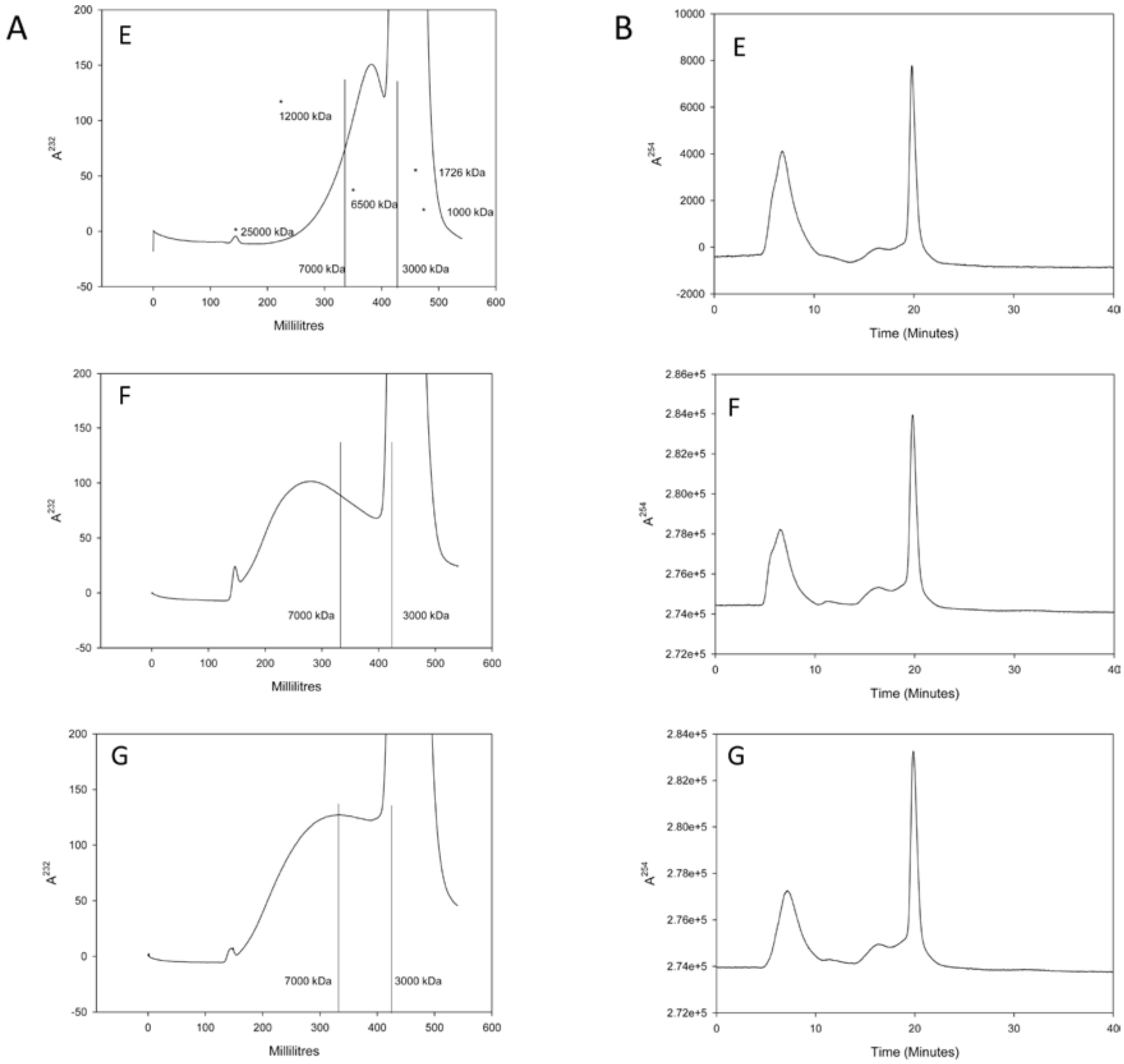

Supplementary Figure S1: Sub-fractionation of the modified heparin derivatives. A. E, F and G were subfractionated on a Sephadex G-100 column (26mm × 1000 mm) in 0.5M ammonium bicarbonate on an AKTA Purifier 10 (GE Life Sciences). Elution was monitored by absorbance at 232 nm. Fractions corresponding to <7000 kDa, ~3000–7000 kDa and <3000 kDa were pooled and freeze dried. Size fractions were determined by calibrating the column using dextran standards as indicated in panel A, and were consistent with previously published calibrations for Sephadex G-100 [3]. The large peak in the <3000 fraction was due to residual copper from the chemical depolymerisation, which was subsequently removed using HiTrap desalting columns (B). The intermediate fractions (~3000–7000 Da) were also desalted using the same method. **B.** Desalting of fractions. The <3000 kDa fractions of heparin-derived polysaccharides E, F and G were desalted to remove residual copper using HiTrap desalting columns (2 × 5 ml) run in HPLC-grade water. Elution was monitored by absorbance at 254 nm. Saccharides elute in the V_0 (at 6 minutes) while copper elutes in the V_T (at 20 minutes). Fractions corresponding to the saccharides were collected, pooled and freeze dried.

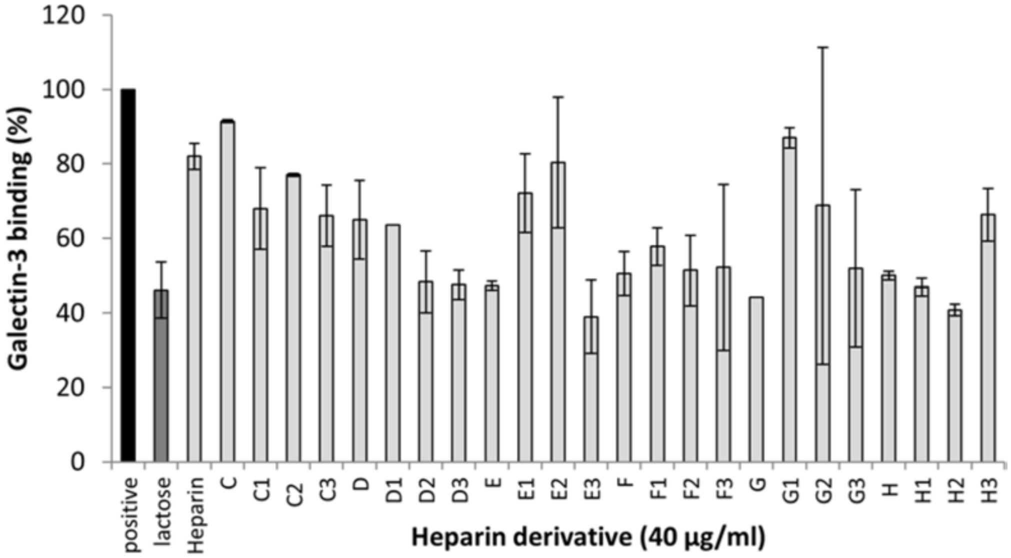

Supplementary Figure S2: Effects of heparin derivatives (40 µg/ml) and their sub-fractions on galectin-3 binding to asialo-fetuin compared to standard heparin (40 µg/ml) and the known galectin-3 inhibitor lactose (40 µg/ml) assessed by galectin-3 ELISA.

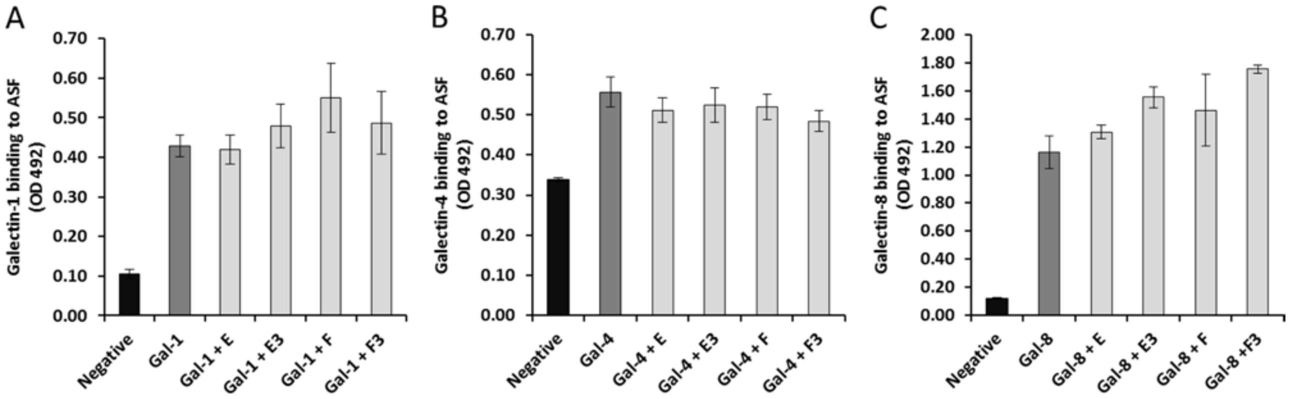

Supplementary Figure S3: None of the potent galectin-3 binding inhibitory heparin derivatives (E, E3, F, F3, 100 µg/ml) shows inhibition of galectin-1 A. −4 B. or −8 C. binding to TF-expressing asialo fetuin (ASF) when assessed by galectin ELISA.

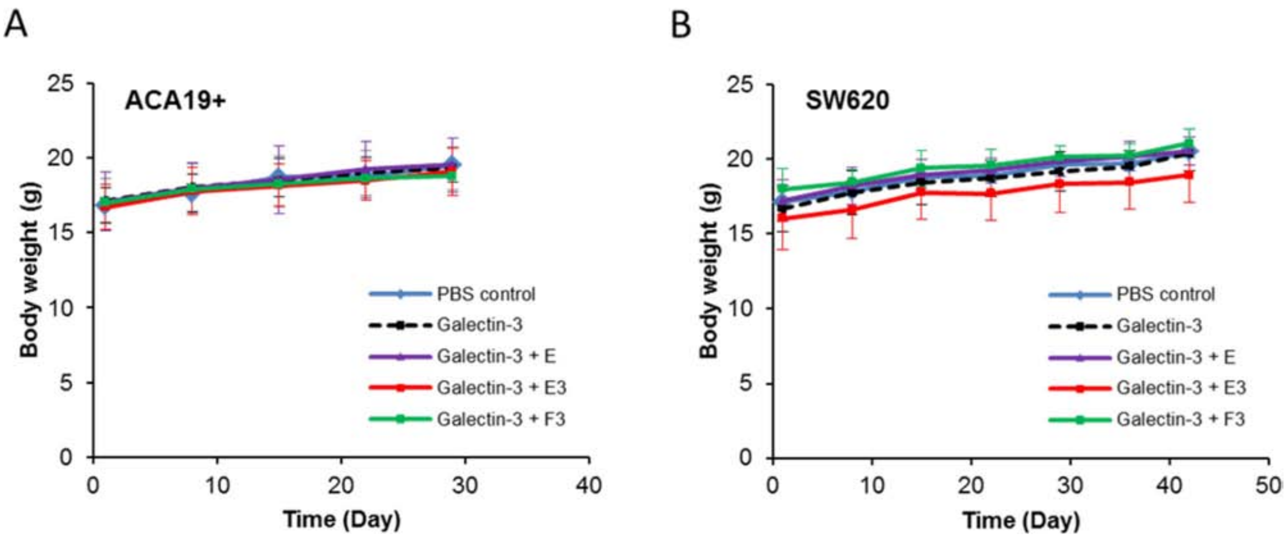

Supplementary Figure S4: Animal body weights of Balb/c nude mice during the course of experiments to assess the effects of modified heparin derivatives on galectin-3 mediated metastasis of human melanoma ACA19+ A. and colon cancer SW620 B. cells. The body weights of mice were measured weekly throughout and mice were sacrificed at 5 weeks (ACA19+) or 6 weeks following tumour cell injection (*n* = 4 for ACA19+ and *n* = 6 for SW620).

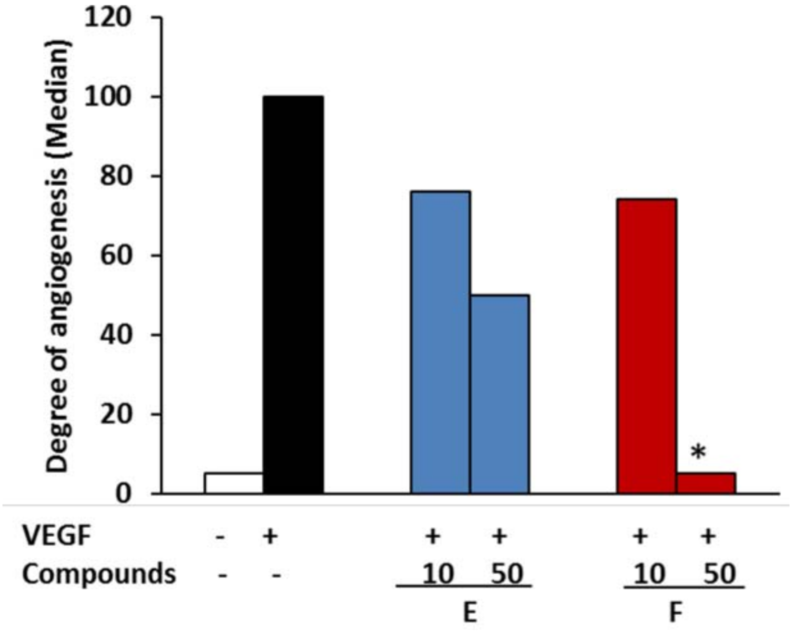

Supplementary Figure S5: Effect of modified heparin derivatives on VEGF-induced angiogenesis *in vivo* in the chick chorioallantoic membrane (CAM) assay. Methyl-cellulose pellets (2 mm diameter) were prepared containing either no or 100 ng of VEGF165, in the presence or absence of 10 or 50 µg of heparin derivative E or F. Samples were applied in the CAM assay on day 8 and the effects measured on day 10, as described previously [4]. Statistical analysis was performed using the Mann–Whitney *U*-test ($*p < 0.05$). Modified heparin derivatives show inhibition of angiogenesis in the presence of VEGF. The compounds alone did not produce any effects on angiogenesis in the absence of VEGF (data not shown).

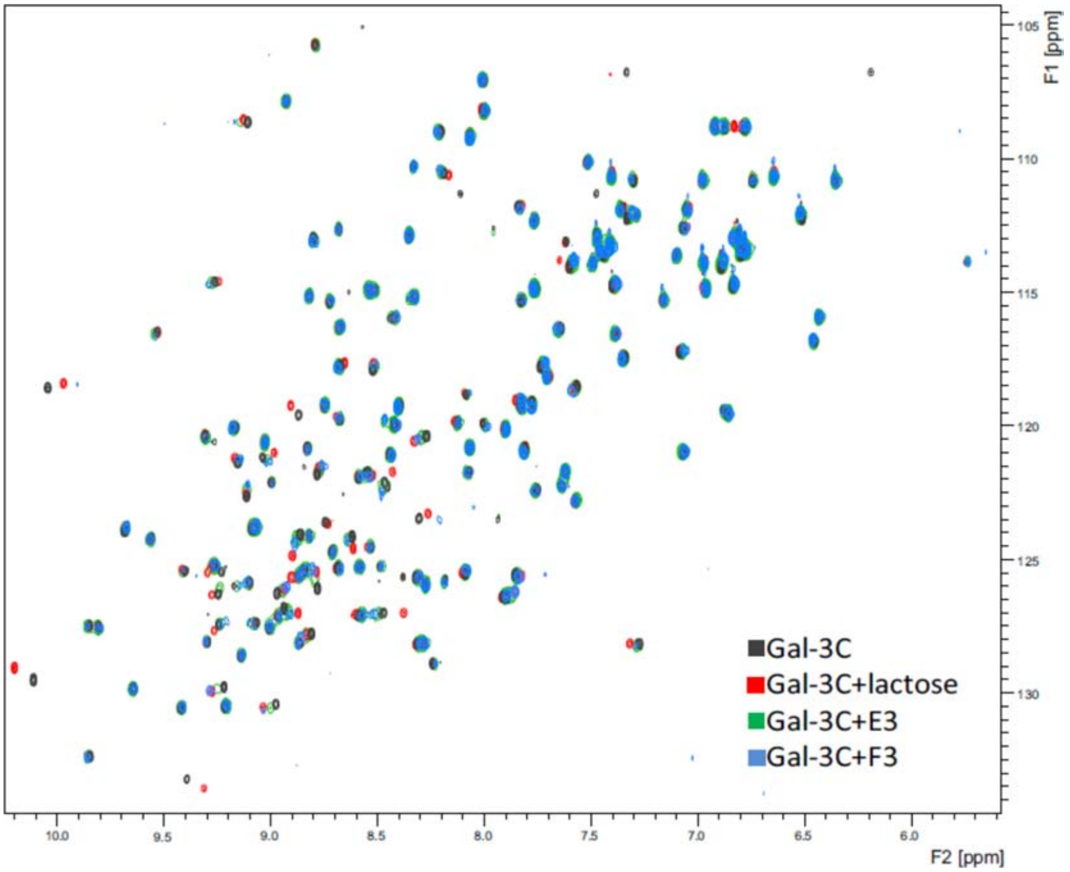

Supplementary Figure S6: NMR analysis of galectin-3 interaction with lactose and the modified heparin derivatives. ^{1}H-^{15}N spectra of Gal-3C in the absence (black), or presence of lactose (red), E3 (green) and F3 (blue) in Gal-3C:ligand ratio of 1:20. Several ^{1}H-^{15}N HSQC cross-peaks show marked chemical shift changes, identifying residues involved in Gal-3C-ligand interaction when compared to Gal-3C alone.

REFERENCES

1. Skidmore MA, Turnbwfull JE. Separation & sequencing of heparin and HS saccharides. In Chemistry and Biology of Heparin and Heparan Sulphate. Ed. H Garg. 2005; 181–203.Elsevier Press.
2. Yates E. A, Santini F, Guerrini M, Naggi A, Torri G, et al. H-1 and C-13 NMR spectral assignments of the major sequences of twelve systematically modified heparin derivatives. *Carbohydrate Research.* 1996; 294:15–27.
3. Laurent TC, Tengblad A, Thunberg L, Höök M, Lindahl U3. (1978). The molecular-weight-dependence of the anti-coagulant activity of heparin. Biochem J. 1978; 175:691–701.
4. West D.C, Thompson W.D, Sells P.G, Burbridge M.F. Angiogenesis assays using the chick chorioallantoic membrane. Methods in molecular medicine - Angiogenesis: Reviews and Protocols. Murray JC. Humana Press. Ch.9. 2001; 107–30.
